# Identification of Novel Hypoxia Subtypes for Prognosis Based on Machine Learning Algorithms

**DOI:** 10.1155/2022/1508113

**Published:** 2022-09-12

**Authors:** Jiawei Wang, Tuo Li, Songquan Wei, Gengye Zhao, Cong Ye, Qiuping Ma, Jinchun Ma, Xiaoyan Cheng

**Affiliations:** ^1^Department of Obstetrics and Gynecology, Nantong Maternity and Child Health Care Hospital Affiliated to Nantong University, Nantong 226018, Jiangsu Province, China; ^2^Department of Endocrinology, Second Affiliated Hospital of Naval Medical University, Shanghai 200003, China; ^3^The Third Affiliated Hospital of Guangzhou Medical University, Guangzhou 510000, China; ^4^Department of Obstetrics and Gynecology, Soochow University Affiliated Taicang Hospital (The First People's Hospital of Taicang), Suzhou, Jiangsu 215400, China

## Abstract

**Objective:**

A reduced level or tension or the deprivation of oxygen is termed hypoxia. It is common for tumours to outgrow their natural source of nutrients, which causes hypoxia in some tumour regions. Hypoxia affects ovarian cancer (OC) in several ways.

**Methods:**

In this study, the expression patterns of prognostic hypoxia-related genes were curated, and consensus clustering analyses were performed to determine hypoxia subtypes in OC included in The Cancer Genome Atlas cohort. Two hypoxia-related subtypes were observed and considered for further investigation. The analyses of differentially expressed genes (DEGs), gene ontology, mutation, and immune cell infraction were performed to explore the underlying molecular mechanisms.

**Results:**

In total, 377 patients with OC were classified into two subgroups based on the subtype of hypoxia. The clinical outcome was considerably poor for patients with hypoxia subtype 2. DEG and protein-protein interaction analyses revealed that the expression levels of *CLIP2* and *SH3PXD2A* were low in OC tissues. Immune cell infarction analysis revealed that the subtypes were associated with the tumour microenvironment (TME).

**Conclusion:**

Our findings established the existence of two distinctive, complex, and varied hypoxia subtypes in OC. Findings from the quantitative analysis of hypoxia subtypes in patients improved our understanding of the characteristics of the TME and may facilitate the development of more efficient treatment regimens.

## 1. Introduction

Ovarian cancer (OC) is by far the deadliest type of gynaecological cancer and the fifth leading cause of cancer-associated death among females [1]. Early diagnosis of OC is challenging owing to the absence of disease-specific symptoms. Subsequently, a majority of women are diagnosed with OC at an advanced stage [2]. Long-term exposure to steroid hormones contributes to several risk factors. Even though hormone synthesis slows down after menopause, women who have been exposed to these hormones continuously and chronically throughout their lives are more likely to develop OC [3, 4]. Currently, the standard-line therapy for OC comprises cytoreductive surgery and chemotherapy (usually paclitaxel and carboplatin) to remove excess tissue [5]. However, even though chemotherapy is occasionally effective in treating early-stage cancer, many patients ultimately develop chemoresistance and experience recurrence [6]. Chemoresistance is driven by molecular and genetic changes that are unknown, and this lack of mechanistic insight hinders its prevention and prediction. [7, 8] Owing to this, novel therapeutic techniques are needed to avoid chemoresistance and increase the success rate of therapy.

Hypoxia is reportedly associated with chemoresistance via several pathways. Altering the metabolism of cancer cells is one of the ways through which hypoxia may cause chemoresistance in patients with cancer. OC cells, when exposed to hypoxia, are subjected to metabolic reprogramming, which alters the glycolytic pathway and enhances resistance to carboplatin [9]. Hypoxia in OC is associated with altered levels of circulating microRNAs (miRNAs), and these miRNA expression patterns are linked to a greater risk of OC development. However, research on the mechanisms underlying hypoxia in OC is insufficient.

Immunotherapy is considered a potentially viable treatment option since it has high degrees of specificity, long-term benefits, and minimal adverse effects. Owing to extensive variability, including clinical and pathological parameters, molecular characteristics, and the immune cell milieu, among other factors, the response rate to immune checkpoint blockade treatment in patients with OC remains as low as 15% [10–12]. Given the heterogeneity of OC, the accurate identification of the specific advantages of immunotherapy in patients is essential for its further advancement [13]. In this study, two distinct hypoxia subtypes were investigated, each characterised by distinct immune infiltrates and immune responses. Additionally, an immune scoring system was developed for patients with OC, which yielded a thorough understanding of the characteristics of the tumour microenvironment (TME) and prompted the development of efficacious treatment modalities.

## 2. Material and Methods

### 2.1. Data Resources

The Cancer Genome Atlas (TCGA, https://cancergenome.nih.gov/) project was used to collect and process the molecular data of 377 individuals who had been diagnosed with OC. The GDC data portal (https://portal.gdc.cancer.gov) was used to obtain the transcriptomic profiles (HTSeq-fragments per kilobase of exon per million mapped fragments (FPKM)) and clinical data for TCGA-OC dataset. The Ensembl IDs were translated to gene symbols, and the FPKM values were transformed into transcripts per million [14].

### 2.2. Identification of Hypoxia Subtypes Using Consensus Clustering

Using the ConsensusClusterPlus tool, subtypes of hypoxia were determined. Hypoxia-related genes are listed in Table 1. To properly classify OC samples, a consensus matrix was developed using consensus clustering. Consistent with the partitioning around the medoids algorithm and using the Pearson correlation coefficient as the distance measure, 500 bootstraps were provided, with each comprising patients with OC included in TCGA cohort. The number of clusters was determined to be two–eight. Additionally, a consensus clustering approach was adopted to classify the genes immunologically related to the prognosis. The consistency matrix and the consistency cumulative distribution function were selected as the methods for optimal classification [15].

### 2.3. Identification of Differently Expressed Genes (DEGs)

The significance analysis built into the empirical Bayes techniques used as a part of the limma package was used to detect DEGs. The cut-off values for selecting the relevant DEGs were a *P*-value <0.01 and a |logFC| > 1. Additionally, using the cBioPortal web platform (https://www.cbioportal.org), we created a network of DEGs and their coexpression genes [16,17].

### 2.4. Gene Ontology (GO) and Pathway Enrichment Analysis

The data were evaluated using functional enrichment analysis to confirm the fundamental function of putative targets. GO is a technique extensively used to annotate genes with functions, including cellular components (CC), biological pathways (BP), and molecular function (MF). ClusterProfiler version 3.18.0 in R was used to examine the GO function of putative targets and enrich the Kyoto Encyclopedia of Genes and Genomes (KEGG) pathway to gain a deeper understanding of how mRNA contributes to the onset and advancement of cancer. The boxplot and heatmap were drawn using the ggplot2 and pheatmap functions of R software, respectively [18].

### 2.5. Mutation Analysis

Using TCGA dataset (https://portal.gdc.com), we retrieved the RNA-seq expression patterns, genetic mutation, and relevant clinical data of 376 patients. The maftools package of R software was used to retrieve data on mutations, which were further visualised by the program. Genes with a higher mutational frequency detected in 376 patients in the histogram are demonstrated.

### 2.6. Protein-Protein Interaction (PPI) Enrichment Analysis

An enrichment study of PPI was performed using the Metascape database for each gene list that was provided. Only the physical interactions observed in the STRING (with a score greater than 0.132) and BioGRID were considered. The resultant network included the subset of proteins that physically interacted with at least one other member in the list. The molecular complex detection (MCODE) algorithm 10 is used to determine the network components that are densely connected when the number of proteins in the network ranges between 3 and 500 [19].

### 2.7. Gene Expression Validation and Survival Analysis of Hub Genes

To further confirm the significant role of hub genes in the pathogenesis and prognosis of OC, we used the Gene Expression Profiling Interactive Analysis (GEPIA) database to retrieve information on the expression of these genes and their prognostic significance. The GEPIA database, an interactive online platform for analysing gene expression, contains data on 8,587 normal samples and 9,736 tumour samples [20].

### 2.8. Cox Analysis

To define the appropriate terms to generate the nomogram, both univariate and multivariate Cox regression analyses were used. Using the “forestplot” R package, we generated a forest plot to display the *P*-value, HR, and 95% confidence interval (CI) for each variable. We created a nomogram based on the findings of a multivariate Cox proportional hazard analysis to accurately predict the 1-year overall recurrence.

### 2.9. Immune Cell Infarction Analysis

We used immuneeconv, an R software package that incorporates the two most recent algorithms, ssGSEA and CIBERSORT, to validate the outcomes of the immune score assessment. These algorithms are benchmarked and have distinct advantages. *SIGLEC15*, *TIGIT*, *PDCD1LG2*, *HAVCR2*, *PDCD1*, *LAG3*, *CTLA4*, and *CD274* were determined to produce transcripts that are important for immune checkpoints, and the expression levels of these eight genes were measured. R foundation for statistical computing (version 4.0.3) was used for implementing the aforementioned analytical techniques. In addition, we used the ggplot2 and pheatmap functions of the R package [21].

### 2.10. Quantitative Reverse-Transcription Polymerase Chain Reaction (qRT-PCR)

Total RNA was extracted from paraneoplastic and tumour tissues of patients with OC using the TRIzol reagent (Sigma-Aldrich, St. Louis, MO, USA). Furthermore, RNA from each sample (2 *μ*g) was subjected to qRT-PCR using the FastStart Universal SYBR ®Green Master (Roche, Germany) on an ABI QuantStudio5 Q5 real-time PCR system (Thermo Fisher Scientific, USA). Afterward, we used cDNA as a template in 20 *μ*L reaction volume (containing 10 *μ*L of a PCR mixture, 0.5 *μ*L of reverse and forward primers, 2 *μ*L of cDNA template, and an appropriate volume of water). We conducted PCR as follows: cycling began with an initial DNA denaturation step at 95°C for 30 s, followed by 45 cycles at 94°C for 15 s, 56°C for 30 s, and 72°C for 20 s. Each sample was assessed in triplicates. Using the 2^−ΔΔCT^ method, readings from the threshold cycle (CT) were obtained and further standardised to the levels of glyceraldehyde 3-phosphate dehydrogenase in each sample. The mRNA expression levels were compared to those in paracancerous tissue controls. The primer pair sequences corresponding to the target genes are presented in Table 2.

## 3. Results

### 3.1. Characterisation of Two Distinct Subtypes of OC Hypoxia

**Figure 1:**
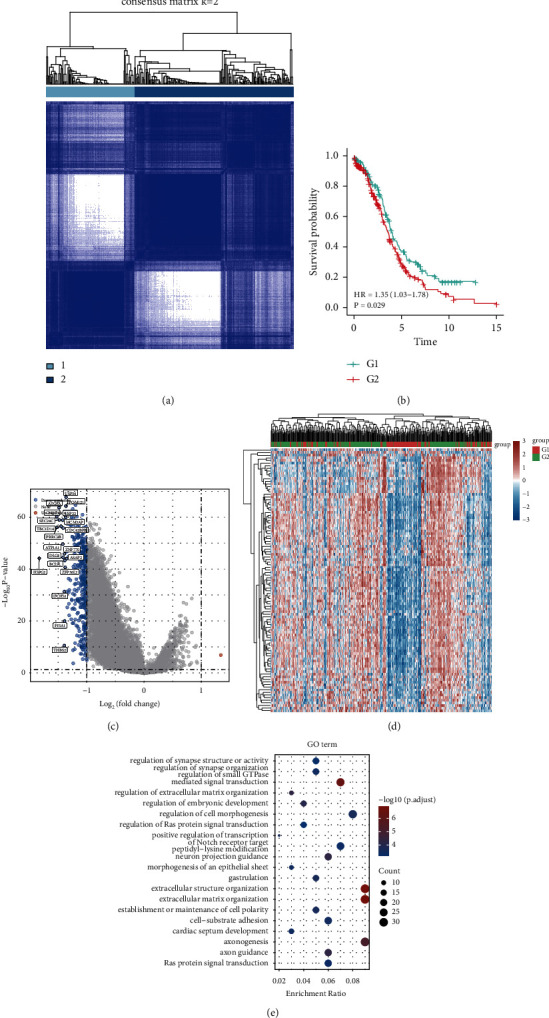
(a) A heatmap illustrating the sample clustering when consensus *k* = 2, based on the expression profile of prognostic immune-related genes. (b) Analysis of survival using the Kaplan-Meier method for the clusters. (c) The fold change values and the P-adjust parameters were used to construct the volcano plot. Upregulated genes are represented by red dots; downregulated genes are represented by blue dots; non-significant genes are represented by grey dots. (d) The heatmap of differential gene expression. (e) The KEGG signalling pathways with significant enrichment illustrate the main biological activities of significant candidate mRNAs. The gene ratio is indicated by the abscissa, and the enriched pathways are indicated by the ordinate. Analysis of putative mRNA targets using the gene ontology (GO) database.

The mRNA expression profiles of hypoxia-associated genes in OC tissues were obtained from the TCGA cohort and used in this investigation. Patients with OC were clustered using consensus clustering methods in line with the expression profiles of prognostic hypoxia-related genes. The stability of clustering was analysed with *k*-values ranging from 2 to 8. As a direct consequence of this, selecting *k* = 2 was the best alternative. Two distinct immune subtypes, immune subtype 1 (*n* = 134) and immune subtype 2 (*n* = 242), were identified in patients with OC. Survival analysis revealed that patients with subtype 2 had a poorer outcome (Figure 1(b)).

### 3.2. Determination of DEGs in Subtypes

The limma program was used to conduct the analysis. The results demonstrated that 375 DEGs, including one gene that was considerably upregulated and 374 genes that were downregulated. The volcano plot of gene expression profile data in each dataset is presented in Figure 1(c). The heatmap of the top DEGs is presented in Figure 1(d).

### 3.3. GO Enrichment Analysis and KEGG Pathways of DEGs

The potential mRNA targets were analysed using the GO database. The findings obtained from the MF, CC, and BP of putative targets clustered, based on the clusterProfiler program in R software, revealed a substantial enrichment of DEGs in functions such as the modulation of synapse structure or activities, modulation of synapse organization, modulation of small GTPase and mediated signal transduction modulation of the extracellular matrix organisation (Figure 1(e)).

### 3.4. Mutation State in Subtypes

**Figure 2:**
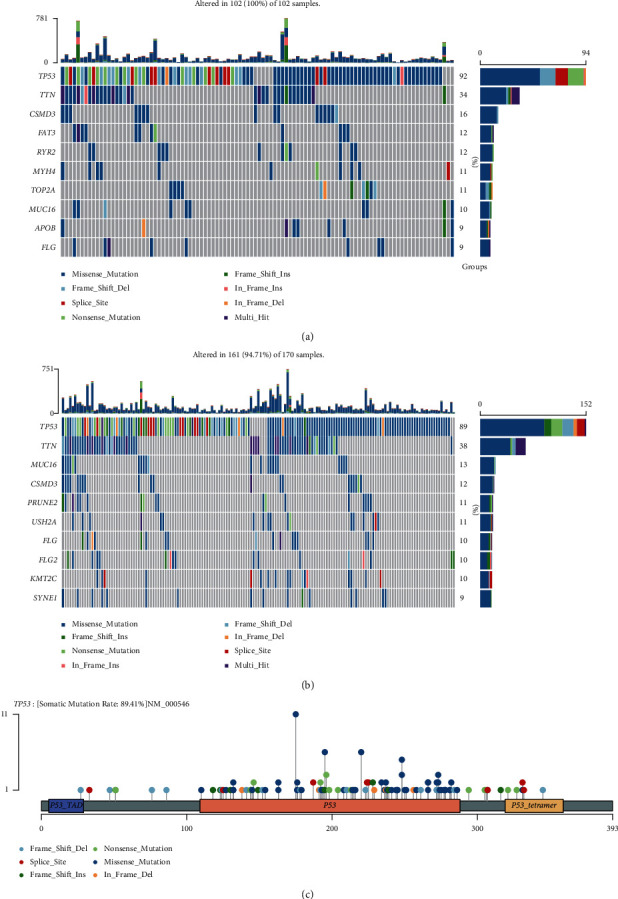
An oncoprint depicting the landscape of somatic mutations observed in ovarian cancer (OC) samples from (a) hypoxia subtype 1 and (b) hypoxia subtype 2. (c) Lollipop charts of the mutated TP53 gene; the figure caption depicts the somatic mutation rate; the subheadings depict the name of the somatic mutation.

We examined how single-nucleotide polymorphisms were distributed among the OC samples. Overall, genetic mutations in immune subtypes 1 and 2 were observed in 102 and 161 OC samples, respectively (Figures 2(a) and 2(b)). Lollipop charts of the mutated *TP53* gene, the figure caption displays the somatic mutation rate, and the subheadings depict the name of somatic mutation (Figure 2(c)).

### 3.5. Establishment of the PPI Network and Module Analysis

**Figure 3:**
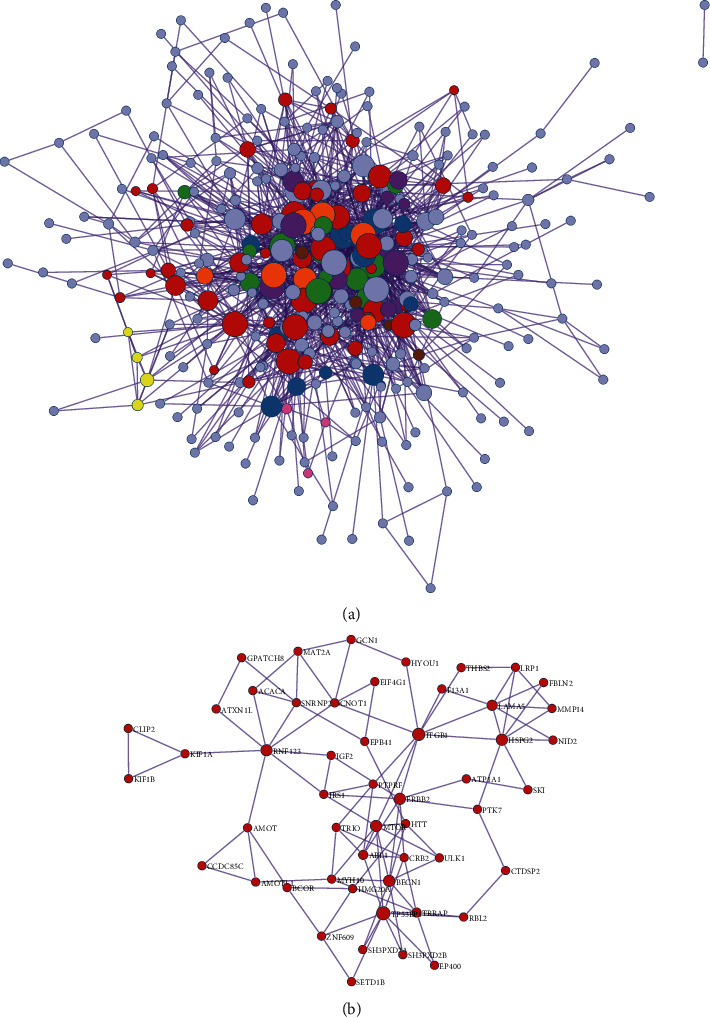
(a) Protein-protein interaction (PPI) network comprising the differentially expressed genes (DEGs) and their co-expressed genes. (b) Hub genes among DEGs.

**Figure 4:**
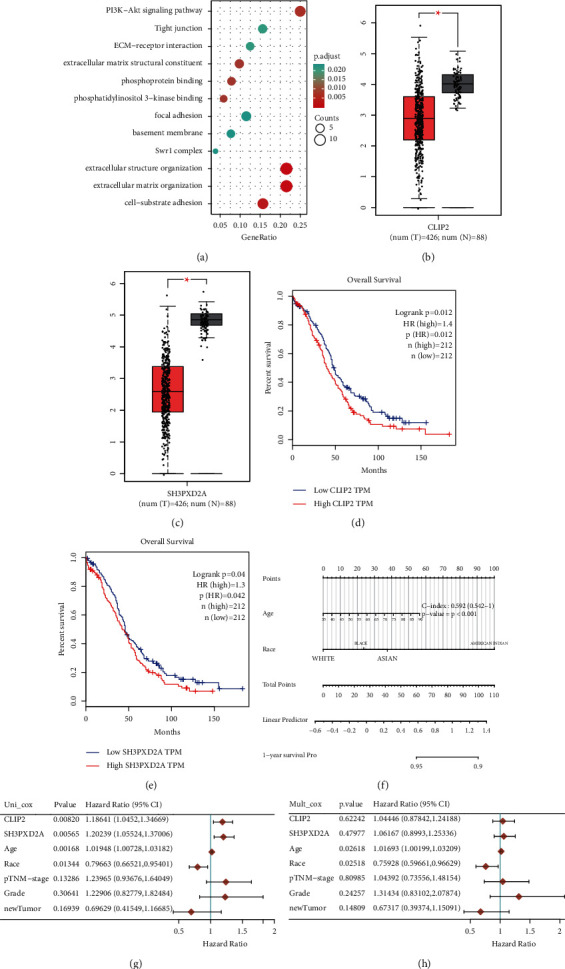
(a) Pop plot of pathway enrichment of hub genes. (b–e) The level of expression of hub genes and the significance of their predictive value based on data from the Gene Expression Profiling Interactive Analysis (GEPIA) database. (f) Nomograms can predict the 1-year overall survival of patients with OV cancer. (g-h) The *P*-value, risk coefficient (HR) and confidence interval analysed by multivariate and univariate Cox regression.

The Metascape database served as the foundation for the establishment of a PPI network of DEGs (Figure 3(a)). The two most significant modules, one comprising upregulated genes and the other comprising downregulated genes, were extracted from this PPI network using MCODE. Hub genes were selected for further analysis. Many hub genes were observed to be enriched in certain pathways, including the PI3K-Akt signalling pathway (Figure 4(a)).

### 3.6. Analysis and Validation of Hub Genes

The screening of the GEPIA database revealed that *CLIP2* and *SH3PXD2A* displayed substantial differences in expression between tumour and normal specimens in OC (Figures 4(b) and 4(c)). The findings of GEPIA for overall survival (OS) revealed that patients with OC were categorised into high- and low-expression groups. We confirmed that the overexpression of *CLIP2* and *SH3PXD2A* was associated with a significantly poor OS in patients.

### 3.7. Survival Analysis

The one-year survival rate for patients with OC may be predicted using the nomogram. We established a calibration curve for the OS based on the nomogram model in the discovery subgroup. The univariate and multivariate analyses showed that *CLIP2* and *SH3PXD2A* expressions functioned independently as a risk factor for OC (Figures 4(d) and 4(e)).

### 3.8. Two Hypoxia Subtypes with Different Immune Infiltrates and Immune Responses

**Figure 5:**
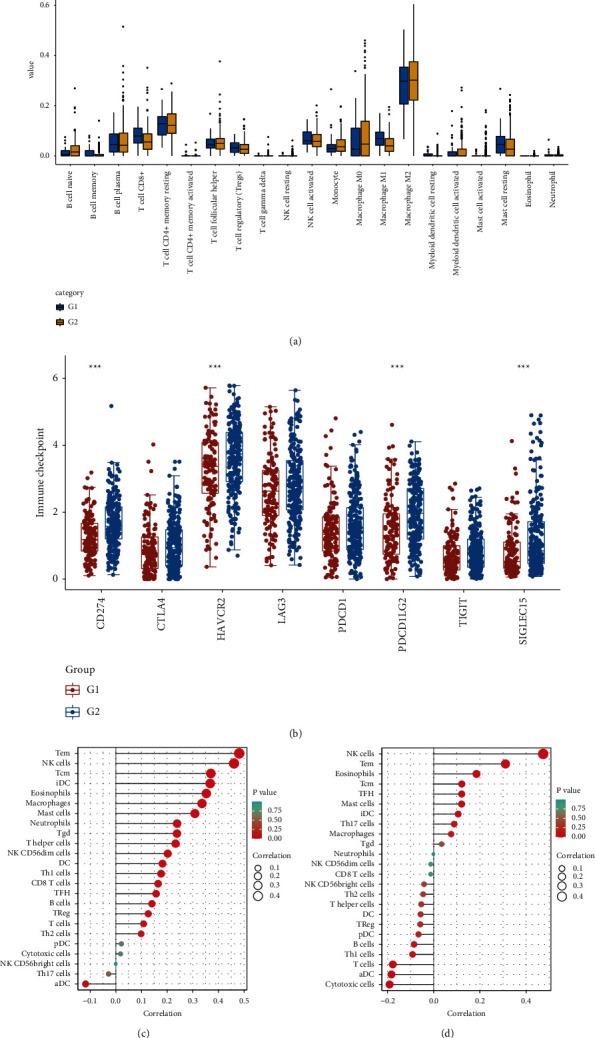
(a) The boxplot of immune infarction cells in hypoxia types 1 and 2. (b) The expression and distribution of immune checkpoint genes in tissues affected by hypoxia types 1 and 2. (c) Barplot of immune cell infarction in high and low *CLIP2* expression obtained via single-sample Gene Set Enrichment Analysis (ssGSEA). (d) Barplot of immune cell infarction of high and low *SH3PXD2A* expression via ssGSEA.

Using the CIBERSORT algorithm, the landscape of tumour-infiltrating lymphocytes was obtained, and 21 types of immune cell profiles of patients with glioma were determined from TCGA. The proportion of cells such as naïve B cells and CD8^+^ T cells differed significantly between the hypoxia subtypes (Figure 5(a)). Checkpoint analysis revealed that hypoxia subtype 2 has a higher expression in C*D274*, *HAVCR2*, *ODCD1LG2*, and *SIGLEC15*(Figure 5(b)). Finally, ssGSEA revealed that *CLIP2* and *SH3PXD2A* expression was positively correlated with immune cells, such as Tem and natural killer (NK) cells (Figures 5(c) and 5(d)).

### 3.9. Evaluation of Gene Expression in OC

**Figure 6:**
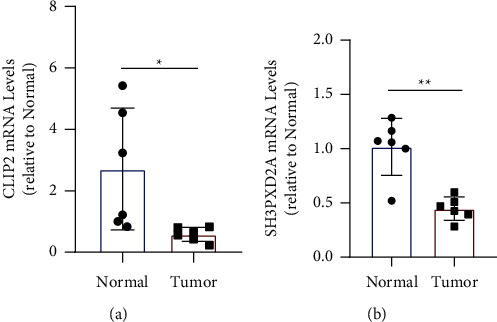
The expression of CLIP2 and SH3PXD2A determined via polymerase chain reaction (PCR).

To validate the expression of the *CLIP2* and *SH3PXD2A* genes in the tumour and nontumour adjacent tissues, the relative mRNA expression levels of *CLIP2* and *SH3PXD2A* in both tumour and nontumour tissues were determined using qPCR. The average expression level of *CLIP2* and *SH3PXD2A* in OC tissue was considerably less than that in normal tissues (Figure 6).

## 4. Discussion

OC is a severe epithelial cancer that predominantly contributes to cancer-associated death among females [22–24]. The treatment options available for OC are clinically ineffective and have a detrimental effect on patients' quality of life. Thus, viable and effective therapies are urgently needed. [25, 26] A growing body of evidence has illustrated that the hypoxia microenvironment plays a critical role in immune response and carcinogenesis based on the dysregulated expression of genes associated with hypoxia. [27, 28] Most research conducted in the past on hypoxia in OV has focused on a single regulator. Hypoxia-induciblefactor-1*α*, for instance, has been reported to play an integral role in various processes, including the promotion of OC immunosuppression, tumour metastasis, and chemoresistance.

In this study, two subtypes of hypoxia were identified using consensus clustering analysis, each of which was based on the prognostic immune-relevant genes. Particularly, hypoxia subtype 2 displayed a more unfavourable clinical outcome than hypoxia subtype 1. Cancer is a malignant neoplasm that may be caused by genetic mutations and variations [29]. Hypoxia subtype 1 was characterised by the presence of more prevalent genetic alterations. Alterations in the expression of several genes, including *TP53*, have been observed to be correlated with the success of immunotherapies and exhibit a predictive potential [30]. In the OC samples, the *TP53* gene was the first to undergo mutation. In hypoxia subtype 1, the *TP53* gene was reported to have a greater incidence of mutations than that in hypoxia subtype 2. Our results suggest a difference among the hypoxia subtypes in terms of genetic changes and mutations.

In this study, we identified 374 genes generated from the hypoxia subtypes, which had the potential to influence pathways such as the PI3K-Akt signalling pathway. Hub genes, such as *CLIP2* and *SH3PXD2A*, were selected and used for further investigation. Recently, the expression of *SH3PXD2A-AS1* was observed to be related to OC; however, the underlying molecular mechanism remains unknown. Simultaneously, the absence of *SH3PXD2A* has been reported in the OV area. Nonetheless, further investigation is warranted. The results of ssGSEA demonstrated that the decrease in *CLIP2* and *SH3PXD2A* expression may influence the infiltration levels of immune cells, such as NK cells. Finally, PCR results confirmed these patterns in OC tissues. While this work is a bioinformatics and pcr analysis, more investigation should be performed in clinic for future application.

A well-defined hypoxia score has significant benefits over standard prognostic markers used for OC. Therefore, the hypoxia score may be used to compare various hypoxia-modulating components and aid the exploration of how tumour cells interact with the immunological milieu. In addition, it helps stratify patients with OC into various groups based on their potential response to chemotherapy or other immune checkpoint blockades. Thus, *CLIP2* and *SH3PXD2A* should be further investigated and could be novel biomarkers for patients with OC.

## Figures and Tables

**Table 1:** Hypoxia-related genes.

Gene symbol	Gene symbol
PSMB6	PSMA1
PSMB5	PSMA8
HIGD1A	PSMC6
EGLN2	PSMD9
PSMD1	RBX1
PSMA7	PSMC5
HIF1AN	PSMB1
PSMC2	PSMB8
PSMD3	PSMA4
EP300	VHL
VEGFA	HIF3A
ELOC	WTIP
PSMC3	EGLN3
PSME4	ARNT
UBE2D2	PSMD12
UBC	PSMA6
PSMD11	EGLN1
PSMD10	PSMB3
PSMB10	PSMD8
PSMD5	CUL2
ELOB	PSMA3
PSME2	PSMC1
CREBBP	SEM1
UBB	EPAS1
PSMD6	PSMA5
PSMD13	PSMA2
PSMB11	EPO
CA9	PSME3
PSMF1	PSMD7
AJUBA	PSMB9
UBE2D1	PSME1
PSMD14	HIF1A
PSMC4	CITED2
PSMB2	UBA52
PSMB4	UBE2D3
LIMD1	PSMD4
PSMD2	RPS27A
PSMB7	

**Table 2:** Primers of CLIP2, SH3PXD2A and GAPDH.

Gene	Forward primer sequence (5′-3)	Reverse primer sequence (5′-3′)
CLIP2	TTAGCGGACAACAGGCTGAC	GCTGGAGCTCCTCGATTTCA
SH3PXD2A	GACTGTACTGCTTAGGGGTGC	CCGCTCTCGTTCTTCTCGAT
GAPDH	AATGGGCAGCCGTTAGGAAA	GCCCAATACGACCAAATCAGAG

## Data Availability

The datasets used and/or analysed during the current study are available from the corresponding author upon reasonable request.

## Abbreviations

OC: Ovarian cancer
TCGA: The Cancer Genome Atlas
DEGs: Differentially expressed genes
TME: The tumour microenvironment
miRNAs: microRNAs
FPKM: Fragments per kilobase of exon per million mapped fragments
TPM: Transcripts per million
GO: Gene Ontology
CC: Cellular components
BP: Biological pathways
MF: Molecular function
KEGG: Kyoto Encyclopedia of Genes and Genomes
PPI: Protein-Protein Interaction
MCODE: Molecular Complex Detection
GEPIA: Gene Expression Profiling Interactive Analysis
CI: Confidence interval
qRT-PCR: Quantitative Reverse-Transcription Polymerase Chain Reaction
NK cells: Natural killer cells
